# A Keyword Analysis Study on Postpartum Obesity Using Big Data

**DOI:** 10.3390/ijerph18168807

**Published:** 2021-08-20

**Authors:** Hyung-ui Baik, Bo-Kyung Seo, Gyu-Ri Kim, Jung-Eun Ku

**Affiliations:** 1 Department of Addiction Rehabilitation and Social Welfare, Eulji University, 553 Sanseong-daero, Sujeong-gu, Seongnam-si 13135, Gyeonggi-do, Korea; smart2ky@eulji.ac.kr (H.-u.B.); 20160722@eulji.ac.kr (B.-K.S.); 2 Department of Beauty and Cosmetic Science, Eulji University, 553 Sanseong-daero, Sujeong-gu, Seongnam-si 13135, Gyeonggi-do, Korea; 3 Department of Beauty Care, Bucheon University, 25 Sinheung-ro 56 Beon-gil, Wonmi-gu, Bucheon-si 14632, Gyeonggi-do, Korea; ku0617@bc.ac.kr

**Keywords:** big data, postpartum obesity, postpartum depression, self-esteem, stress, maternal diet data

## Abstract

This study selected Google and Naver, the most recognizable Internet portals in Korea, as subjects for analysis. “Postpartum obesity” and “postpartum depression” were used as keywords for data collection. This study aimed to provide basic data for solving maternal problems using big data. Keywords related to postpartum obesity were collected from the portal site Google from 1 January 2019 to 31 December 2019. The collected data were analyzed through simple frequency analysis, N-gram analysis, and keyword network. This study can be used as basic data for postpartum obesity-related programs or academic research. It is also expected to be used for research on the development of a mobile-based customized healthcare system focused on maternal health. Previous papers and data are still insufficient at solving the physical and mental problems related to postpartum obesity and depression. It is necessary to find ways to continuously integrate and collect data from mothers across the country.

## 1. Introduction

Pregnancy and childbirth, which cause weight gain, may lead to obesity after delivery and thus be a risk factor for disease. Weight gain due to pregnancy is an important health indicator; it causes complications not only during the prenatal period and delivery but also regarding breast cancer, heart disease, and diabetes after childbirth. Postpartum weight gain may also lead to decreased self-esteem, depression, binge eating, decrease in parenting ability, and psychological problems. Thus, the need for prenatal and postpartum obesity management is becoming more prominent [1].

The average weight retention after childbirth, which concerns many women, is approximately 1–1.5 kg. When this is corrected, considering normal weight gain and weight measurement errors due to age, the effect of pregnancy itself is likely to be only approximately 0.5 kg. However, approximately 15–20% of pregnant women experience significant postpartum weight gain, and at least 10% experience significant retention of approximately 7 kg [2].

Women have specific reproductive and medical characteristics and undergo major changes during menstruation, the postpartum period, and menopause. Pregnancy and childbirth cause many changes in a woman’s body; this can influence weight changes in the future and can possibly cause obesity. In fact, among patients visiting the obesity clinic, women who have recently given birth often attribute their obesity to pregnancy and childbirth [3], with 89% of women experiencing postpartum obesity [4]. Mothers ultimately complain of various physical and mental pains after childbirth, including postpartum obesity. This causes internal diseases and various mental health challenges by causing mothers to avoid working and provoking interpersonal discomfort due to a sudden change in appearance. Mothers with postpartum obesity obtain information from the Internet, wherein they can easily and quickly obtain and apply them, such as when trying a diet. The Internet is an essential source for learning because it is easy to access, has no restrictions on time and place, and contains a lot of information [5,6]. If the mother is within a normal range in terms of body mass index, gaining about 11.5 to 16 kg of weight during pregnancy is adequate. However, it is recommended to maintain a weight gain of 7 to 11.5 kg for overweight mothers and 5 to 9 kg for obese mothers. Weight loss after pregnancy is undesirable and may be associated with low birth weight [7]. Postpartum obesity is defined as when the mother’s weight does not return to its pre-pregnancy level even after six months after childbirth and when her weight has increased by 2.5 kg or more. Among the factors that cause postpartum obesity, gestational weight gain during pregnancy and postpartum weight retention at six months after childbirth are presented. After six months after childbirth, the increased weight is fixed as is. Therefore, it is very difficult to control the weight, so it is better to control the weight within six months after childbirth. Failure to lose weight within six months of giving birth increases the likelihood of long-term obesity. Therefore, a safe, long-term, and early weight control strategy is needed. Diet, moderate exercise, and breastfeeding are the most effective ways to reduce body fat [8].

This study can be used as basic data for postpartum obesity-related programs or academic research. This study is expected to be used for research on the development of a mobile-based customized healthcare system for the health of pregnant women.

## 2. Materials and Methods

### 2.1. Subjects and Duration

This study collected data from Naver, the most recognized portal site in Korea, for postpartum obesity analysis. The collection period was from 1 January 2019 to 31 December 2019. The Python 2.7 (Pycon, Python Software Foundation, Delaware, USA) program was used for data collection. Sentences containing the keywords “postpartum obesity” and “postpartum depression” were collected from Naver News using the function of the Naver Search Application Programming Interface. A total of 452 news pages containing the keywords were obtained.

Among the patients who visited the obesity clinic, women who have given birth often consider pregnancy and childbirth to be the cause of obesity. According to reports, 89% of women suffer from obesity after childbirth.

Postpartum obesity refers to cases wherein body weight increases by more than 2.5 kg from pre-pregnancy weight and persists six months after childbirth. Regarding postpartum obesity, there are no clear regulations regarding its definition, terminology, or timing. Therefore, this study also investigated foreign papers that included expressions such as “postpartum weight retention” [9].

### 2.2. Simple Frequency Analysis

In order to understand the contents of the large amount of collected data [10], analyzing the frequency of the appearance of keywords related to the subject keyword in a hierarchical order is useful. This is because the presence of related keywords can reflect an interest in the subject keyword [11]. For data collection, the Python 2.7 (Pycon, Python Software Foundation, Delaware, USA) program was utilized. In addition, sentences containing the keyword “postpartum diet” were collected from Naver blogs, web documents, news, and cafes using the Naver Search Application Programming Interface (API). The collected sentences were then processed using the Mecab (Unjeon, Korea) program, which performs a morphological analysis according to Korean characteristics with minimal changes to the project. After that, nouns related to postpartum obesity and health were extracted. We analyzed the common keywords that appeared in the collected data through a simple frequency analysis.

### 2.3. N-Gram Analysis

Although a simple frequency analysis can examine the frequency of the entire dataset, this cannot identify the simultaneous occurrences and density of the subject keywords and related keywords. Thus, this study conducted an N-gram analysis by separating the data into keyword units. Keywords 1 and 2 of the N-gram were also used to confirm the direction between the keywords. N-gram analysis, which can analyze the frequency of simultaneous appearances through keywords and the degree of density between them, was performed. In Table 2, the words that appeared simultaneously with the keyword “depression” are as follows: “postpartum” appeared 570 times, “prevention” appeared 62 times, “counseling” appeared 44 times, and “treatment” appeared 42 times.

### 2.4. Keyword Network Analysis

Keyword network analysis is used to study the connection and relationship between keywords by looking at the forms of nouns and adjectives in a sentence. To perform this, the data must first be implemented in the form of a social matrix. For this purpose, Textom, a social matrix program provided by the IMC (Integrated Marketing Communication), was used in this paper. For the keyword network analysis itself, CONCOR (CONvergence of iteration CORrealtion) analysis was used, and the Ucinet 6 program (Analytic Technologies, Nichollasville, KY, USA) was used to visually represent this.

## 3. Results

### 3.1. The Result of Simple Frequency Analysis

To determine the keywords related to postpartum depression and obesity, the frequency of occurrence of keywords was examined. Table 1 is a frequency table showing the top 20 keywords that appeared in relation to the keyword search (“postpartum depression” and “postpartum obesity”). The keywords that appeared frequently were as follows: “depression” (1247), “childbirth” (1178), “postpartum” (1068), and “health” (804). Hence, these keywords are related to postpartum and postpartum obesity. The top 21–30 keywords included “families” (314), “counseling” (257), and “exercise” (249).

### 3.2. N-Gram Analysis Result

By performing N-gram analysis, the frequency of simultaneous appearances was analyzed through the degree of concentration between keywords. In the word separation stage, postpartum depression was divided into “postpartum” and “depression”, and postpartum obesity was divided into “postpartum” and “obesity. Therefore, words were compared with keywords for depression and obesity.

As shown in Table 2, the words that appeared simultaneously with the keyword for depression were “postpartum” (570), “prevention” (62), “counseling” (44), and “treatment” (42). During simple frequency analysis, each word was ranked according to frequency. “Postpartum” was in third place, “prevention” was 48th, “counseling” was 28th, and “treatment” was seventh. Furthermore, as shown in Table 2, the following keywords appeared simultaneously with “obesity”: “postpartum” (81), “management” (22), and “treatment” (21). On simple frequency analysis, “postpartum” ranked third, “management” was 12th, and “treatment” was seventh.

**Figure 1:**
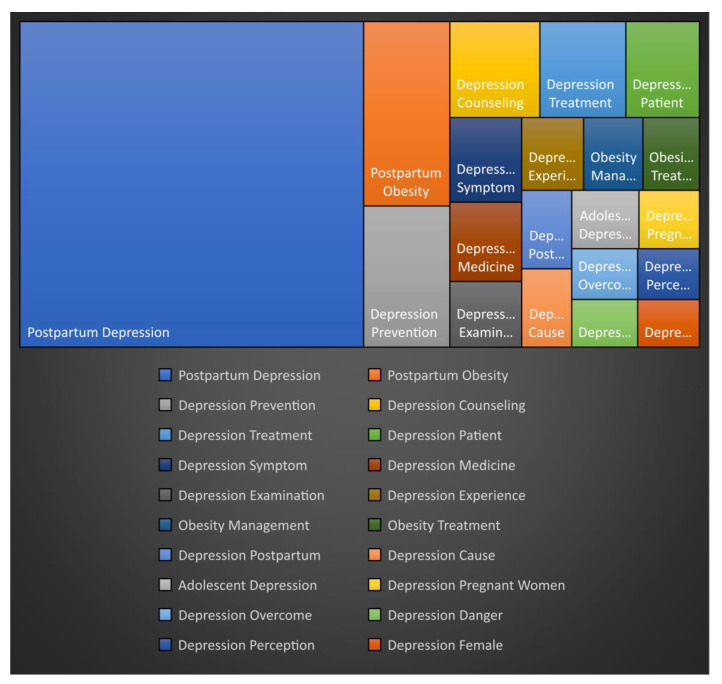
Frequency of keywords by N-gram analysis.

As shown in Figure 1 and Table 2, using Keywords 1 and 2, the relationship between keywords becomes evident. For example, since “depression” and “obesity” appear after “postpartum”, it can be confirmed that the interest in depression and obesity is high after childbirth. Moreover, since “depression” is followed by the keywords “prevention”, “counseling”, and “treatment”, we can confirm a high degree of interest in treatment and prevention methods for depression. Lastly, because “management” and “treatment” follow the keyword “obesity”, we can also confirm a high degree of interest in the management methods for obesity.

### 3.3. Keyword Network Analysis Result

**Figure 2:**
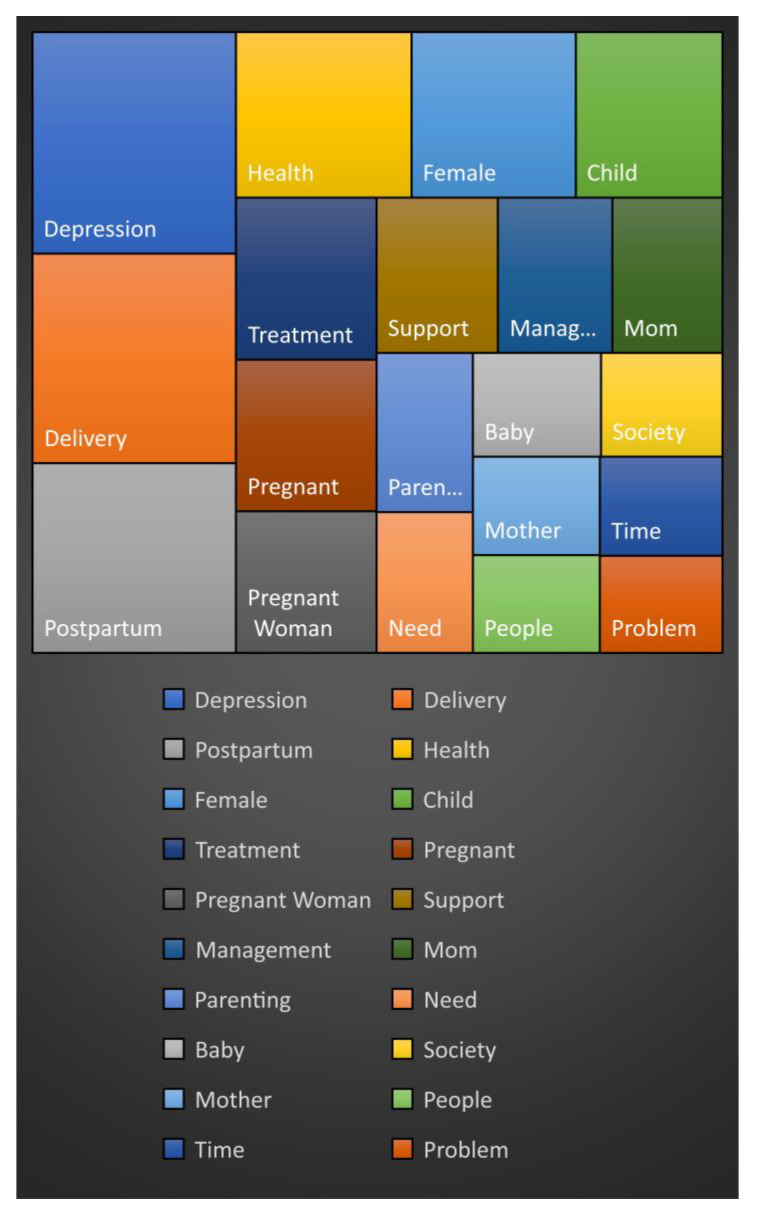
Frequency of keywords using the simple frequency analysis.

CONCOR analysis was applied for the keyword network analysis. Figure 2 represents the result of the CONCOR analysis, and the data were classified into four groups.

For the postpartum care group, the keywords “postpartum care”, “postpartum care center”, “family”, “health”, “pregnant women”, “couples”, “newborn babies”, “children”, “public health centers”, “mental health”, and “safety” were grouped together. For the government program group, the keywords “government”, “information”, “participation”, “planning”, “education”, and “application” were grouped. For the health concern group, the keywords “depression”, “diet”, “stress”, “disease”, “suicide”, “obesity”, “risk”, “recovery”, “treatment”, “prevention”, “improvement”, “exercise”, “effect”, “hospital”, “patient”, “expert”, “counseling”, “symptoms”, and “cause” were grouped. For the family member group, the keywords “family”, “parents”, “mothers”, “fathers”, “wives”, “husbands”, “babies”, “parenting”, “offspring”, “child”, “children”, “childbirth”, “happiness”, and “marriage” were grouped together.

## 4. Discussion

It is important to discuss the limitations of postpartum depression research that previous studies have pointed out. First, there are no definitive data on the factors related to postpartum depression. Second, research data on mental and physical health are insufficient. Therefore, many researchers recommend that, to overcome this limitation, sufficient data to predict factors influencing depression must be collected. Additionally, by accurately differentiating between general female obesity and maternal obesity, measures can be taken so that general maternal women do not manage obesity in a dangerous way according to misleading advertisements on the Internet. Lastly, on the basis of the results of big data analysis, mothers should be able to obtain useful information and to perform more extensive research in the future so that they are not exposed to risk [12]. In future research, we plan to collect various maternal healthcare data related to “untact” in the post-COVID-19 era (Coronavirus disease-2019). In addition, we want to study using more innovative technologies and algorithms. Maternal healthcare data are consistent in its aspect but is changing rapidly due to people’s interests. In particular, it is necessary to more accurately identify and analyze promotional maternal healthcare that changes every moment and is sensitive to trends. For this, periodic monitoring borders require continuous monitoring and analytical research.

## 5. Conclusions

This study applied a big data analysis method [13,14] instead of a questionnaire-based survey to determine the trends and perceptions of people with postpartum obesity in Korea. Simple frequency analysis, N-gram analysis, and keyword network analysis were performed by analyzing sentences containing diet keywords from 1 January 2019 to 31 December 2019, through the portal site Naver. This study can be used as basic data for postpartum obesity-related programs or academic research. Furthermore, this data can help develop a mobile-based customized health management system program for the health of pregnant women [15,16,17].

In this study, sentences containing the keywords “postpartum obesity” and “postpartum depression” were collected from Naver News. Using the collected data, simple frequency analysis, N-gram analysis, and keyword network analysis were performed. Simple frequency analysis cannot analyze the density of keywords by simply listing the frequency of occurrence of all data. Thus, the results were further analyzed using N-gram analysis, a concept similar to the automatic completion function of the search word in portal sites.

In the simple frequency analysis, the keyword “depression” appeared the most (1247 times). Using N-gram analysis, it was confirmed that the keyword “depression” was searched along with “prevention”, “counseling”, and “treatment”; this implies that people have a high interest in treating depression. Moreover, on simple frequency analysis, “birth” appeared 1178 times, taking second place, whereas “health” appeared 804 times, taking fourth place. Interpreting this result in relation to the result of the N-gram analysis, people are very interested in the management and treatment of the symptoms of postpartum obesity and depression.

Through the keyword network analysis, the groups between keywords were divided into four categories according to group characteristics: the postpartum care group, government project group, health concern group, and family member group.

This study does not claim that the proposed theoretical model fully explains all factors related to maternal health. However, it provides insight into postpartum health by simultaneously showing the dynamic relationship between the mother, baby, and environmental factors. Additionally, this study comprehensively deals with the correlations between postpartum obesity and depression, which have been discussed only to a limited extent in previous studies. Additionally, in terms of research design, various measurement tools [18] with high explanatory power, reliability, and validity were used for theoretical variables, breaking away from existing research designs that collected data with only one question and did not use validated tools. As the big data analysis research design was able to comprehensively measure keywords related to postpartum obesity [19,20], our findings can be used as basic data for postpartum obesity-related programs or academic research. We hope our data can be useful in research [21,22] for the development of a novel, mobile, customized health management system for maternal health.

## Figures and Tables

**Table 1:** Frequency of keywords by simple frequency analysis.

Rank	Keyword	Frequency
1	Depression	1247
2	Delivery	1178
3	Postpartum	1068
4	Health	804
5	Female	752
6	Child	671
7	Treatment	629
8	Pregnant	589
9	Pregnant Woman	550
10	Support	521
11	Management	491
12	Mom	472
13	Parenting	424
14	Need	375
15	Baby	367
16	Society	348
17	Mother	346
18	People	343
19	Time	336
20	Problem	329

**Table 2:** Frequency of keywords using the N-gram analysis.

Rank	Keyword 1	Keyword 2	Frequency	Rank	Keyword 1	Keyword 2	Frequency
1	Postpartum	Depression	570	11	Obesity	Management	22
2	Postpartum	Obesity	81	12	Obesity	Treatment	21
3	Depression	Prevention	62	13	Depression	Postpartum	20
4	Depression	Counseling	44	14	Depression	Cause	20
5	Depression	Treatment	42	15	Adolescent	Depression	20
6	Depression	Patient	36	16	Depression	Pregnant Women	18
7	Depression	Symptom	31	17	Depression	Overcome	17
8	Depression	Medicine	29	18	Depression	Danger	16
9	Depression	Examination	24	19	Depression	Perception	16
10	Depression	Experience	23	20	Depression	Female	15

## Data Availability

Not applicable.
